# Informing Patient Relatives in Intensive Care Units… Face to Face or by Phone?

**DOI:** 10.3390/healthcare14132026

**Published:** 2026-07-07

**Authors:** Tuba Çatak, Elif Kerimoğlu, Ali Özgül Saltalı

**Affiliations:** 1 Department of Pediatric Dentistry, Anesthesiology and Reanimation, Faculty of Dentistry, Necmettin Erbakan University, Beyşehir Street, 42090 Konya, Turkey; 2 Department of Intensive Care, Training and Research Hospital, Ordu University, 52200 Ordu, Turkey; 3 Department of Anesthesiology, Konya Numune Hospital, Hastane Street, 42060 Konya, Turkey

**Keywords:** intensive care units, communication, family-centered care, family satisfaction

## Abstract

**Background**: Effective communication with relatives is an essential component of family-centered intensive care. This study evaluated preferences for receiving information in intensive care units (ICUs) and examined factors associated with communication expectations and perceived adequacy of information. **Methods**: In this cross-sectional survey, 401 participants completed a 25-item questionnaire developed from the literature, reviewed by experts, and pilot-tested. Communication preferences and perceptions were analyzed according to age, sex, education, marital status, geographic region, employment status, healthcare background, degree of kinship, duration of ICU stay, and previous ICU experience. Multivariable logistic regression was performed to identify factors independently associated with considering telephone-based communication sufficient. **Results**: Most participants (89.8%) preferred face-to-face communication. Overall, 62.8% considered telephone-based information insufficient, and face-to-face communication was more frequently perceived as promoting trust and understanding. Communication preferences varied according to demographic, educational, regional, and experiential characteristics. Women placed greater importance on direct interpersonal communication, whereas men favored more practical information delivery. Participants with higher educational attainment reported greater expectations regarding the structure and content of information. In multivariable analysis, male sex (OR = 2.63, 95% CI: 1.40–4.93; *p* = 0.003), residence outside the city where the hospital was located (OR = 2.86, 95% CI: 1.49–5.46; equivalent to OR = 0.35 for residence within the hospital city; *p* = 0.002), and having a relative hospitalized in the ICU for 21–31 days (OR = 3.74, 95% CI: 1.42–9.86; *p* = 0.008) were independently associated with considering telephone-based communication sufficient. **Conclusions**: Face-to-face communication was the preferred method of information delivery for most relatives. Communication preferences and the perceived adequacy of telephone-based information varied according to demographic, regional, and clinical characteristics. These findings support adopting individualized communication strategies rather than a uniform approach when communicating with relatives of intensive care patients.

## 1. Background

Intensive care units (ICUs) require multidisciplinary teamwork to provide advanced care to patients whose lives are under threat [1]. Patients hospitalized in these units typically face an uncertain length of stay and a high risk of morbidity and mortality [2]. This situation can lead to intense stress, anxiety, and feelings of helplessness among patients’ relatives [3,4]. Molter identified the fundamental needs of relatives of intensive care patients as information, trust, support, and proximity to the patient. Meeting these needs increases families’ capacity to cope with stress and enhances overall satisfaction levels [5]. Understanding the needs of patients’ relatives facilitates effective communication with healthcare personnel and improves patient care outcomes [6].

However, research on the effects of cultural and regional factors on the anxiety and satisfaction levels of relatives of intensive care patients is limited [7]. This study is based on the hypothesis that communication preferences, perceived adequacy of information, and information-related needs of individuals whose relatives are hospitalized in the ICU may vary according to sociodemographic characteristics. Although communication in intensive care units has been extensively studied, relatively little is known about how sociodemographic and regional factors influence family members’ preferences for face-to-face versus telephone-based communication. In culturally diverse settings such as Turkey, these determinants remain insufficiently explored. The primary aim of the study was to evaluate relatives’ preferences for the mode of information delivery (face-to-face or telephone) together with their perceived adequacy of communication and information-related expectations. Secondly, the moderating effects of variables such as geographic area, education, age, marital status, sex, and degree of kinship on this relationship were examined. In addition, participants’ expectations and recommendations for physicians were analyzed according to their sociodemographic characteristics. The findings are intended to contribute to the standardization of information processes in intensive care units and to the development of approaches that are sensitive to families’ demographic and cultural characteristics.

## 2. Materials and Methods

### 2.1. Study Design

This study is a descriptive, comparative, cross-sectional survey.

### 2.2. Ethical Considerations

The study was approved by the Necmettin Erbakan University Faculty of Dentistry Non-Drug and Non-Medical Device Research Ethics Committee, on 26 December 2024 (Turkey/Konya; Decision No. 2024/525; ID: 22514). The study was conducted in accordance with the principles of the Declaration of Helsinki. All participating units and personnel were informed about the study procedures. Participation was voluntary, and participants’ identities were anonymized. Participants were informed that they had the right to withdraw from the study at any stage. The first question of the survey asked whether they agreed to participate voluntarily. Only those who answered “yes” to this question were allowed to continue completing the survey.

### 2.3. Data Collection Instruments and Procedures

This study was conducted using a structured questionnaire administered to relatives of patients in intensive care units (ICUs). A total of 401 participants were included using a non-probability sampling strategy. Participants were recruited through face-to-face interviews conducted in the ICUs of two hospitals and through an online questionnaire administered to eligible relatives who lived in distant locations or were unable to attend the hospital during the study period. Because the target population was not equally accessible, probability-based sampling methods, such as random or consecutive sampling, were not considered feasible. The 25-item questionnaire, developed based on the literature, began with an item querying voluntary consent; only those who responded “Yes” proceeded to the remaining items. The questionnaire was prepared in Turkish and translated into English only for publication purposes (Supplementary Materials File S1). A screenshot of the first page of the face-to-face survey questionnaire is provided in Supplementary Materials File S2. In addition, an anonymized dataset containing participants’ responses to the survey questions is provided in Supplementary Materials File S3 to support transparency and verification of the study findings. Face validity was assessed by two anesthesiology specialists and one intensive care subspecialist. Based on their feedback, the questionnaire was revised to improve clarity and comprehensibility. A pilot study was then conducted with 30 individuals, and additional minor modifications were made to improve the flow and readability of the questionnaire. Administration was carried out face-to-face or online by pre-trained researchers, with standardization maintained throughout the data collection process. Not all questionnaire items were included in the present report. Analyses focused on items directly related to communication preferences, perceived adequacy of information, and relatives’ needs during the ICU process. Several exploratory items were collected for descriptive purposes and are not presented in detail in this manuscript.

The item “Suggestions to physicians” was included as an optional open-ended question. Therefore, participants were not required to provide a response, and analyses of this item were performed only among respondents who answered the question.

### 2.4. Participants

This study was conducted through face-to-face interviews or via an online survey (Google Forms) with the relatives of patients hospitalized in intensive care units. Both formats used identical questionnaires and standardized instructions. Participants’ identities were anonymized, and the first question of the survey asked whether they agreed to participate voluntarily. Only those who answered “yes” to this question were allowed to continue completing the survey. Participants aged 18 years or older who were able to read or understand Turkish and who voluntarily agreed to participate were eligible for inclusion. Individuals younger than 18 years of age, those unable to understand Turkish, and questionnaires with substantial missing data were excluded from the analysis. Face-to-face data were collected from relatives of patients admitted to the intensive care units of Konya Numune Hospital and Ordu State Hospital. A total of 250 participants were recruited through face-to-face interviews. The remaining participants completed the identical questionnaire online via Google Forms. The online survey was specifically intended for relatives living in distant geographical locations or for those who were unable to attend face-to-face interviews during the study period. The same questionnaire and standardized instructions were used for both data collection methods to ensure methodological consistency.

### 2.5. Sample Size and Power Analysis

An a priori power analysis for chi-square contingency testing was conducted. Based on a medium effect size (w = 0.30), 95% statistical power (1 − β = 0.95), and a 5% margin of error (α = 0.05), the minimum required sample size was calculated as 365 (Supplementary Materials File S4). To account for potential data loss or missing responses, an additional 10% was added, and the target sample size was set at 401. During data collection, responses from all 401 participants were obtained, and the study achieved the intended power (actual power = 97.0%). To further evaluate the adequacy of the sample size for the multivariable logistic regression model, an events-per-variable (EPV) assessment was performed. The binary outcome (considering telephone-based communication sufficient) occurred in 149 participants. The final model included six candidate predictors. Because two predictors were categorical variables with more than two levels, the number of dummy-coded parameters was also considered. Accordingly, the model included 13 predictor parameters, yielding an EPV of 149/13 = 11.5. This value exceeds the commonly recommended minimum threshold of 10 events per parameter, supporting the adequacy of the sample size for the fitted logistic regression model. All sample size and power calculations were performed using G*Power software (version 3.1.9.7, Heinrich-Heine-Universität Düsseldorf, Düsseldorf, Germany).

### 2.6. Pilot Study and Statistical Analysis

A 25-item questionnaire was administered to individuals with relatives receiving treatment in intensive care. A pilot study with 30 participants was conducted to evaluate comprehensibility, duration, and item clarity. Based on participant feedback, wording was simplified, item order was revised, and administration time was optimized. Pilot data were used solely to assess face validity and comprehensibility and were not included in the main analyses. Internal consistency analysis was not performed because the questionnaire was designed to collect information across multiple independent domains rather than to measure a single underlying construct using multiple items. Therefore, internal consistency was not considered an appropriate psychometric property for this questionnaire. Face validity and comprehensibility were assessed through expert review and pilot participant feedback. The final questionnaire included sections on sociodemographic characteristics, place of residence and employment status, intensive care experience, methods of obtaining information, perceived adequacy of communication, information preferences, recommendations to physicians, and basic needs.

Descriptive statistics (frequency, percentage, mean, median, standard deviation) were calculated. Relationships between variables were examined using cross-tabulations and the Chi-square test. When expected cell counts were low, Fisher’s Exact Test was used, and Monte Carlo estimations were applied when appropriate. The significance level was set at α = 0.05, and results were reported as *p* < 0.001, *p* < 0.01, and *p* < 0.05. All variables that were significantly associated with perceived sufficiency of telephone-based communication in univariable analyses were included as candidate predictors and entered into the multivariable logistic regression model using the enter method. Because the subgroup analyses were exploratory in nature, no formal adjustment for multiple comparisons was applied. Accordingly, subgroup findings should be interpreted with appropriate caution. Wherever possible, exact *p* values are reported to improve the transparency of statistical reporting.

All analyses were performed using IBM SPSS 24.0, Jamovi 2.4, Microsoft Excel 2016, and RStudio 2023.09.0.

## 3. Results

### 3.1. Main Findings of the Study

A large majority of participants (89.8%) preferred face-to-face communication and reported that this method improved their understanding of the information and enhanced perceived trust. In a separate assessment, 62.8% of participants considered telephone-based communication inadequate. Because communication preference and perceived adequacy represent distinct but related outcomes, they were analyzed and interpreted separately throughout the study. To evaluate the potential influence of survey mode, a sensitivity analysis comparing face-to-face and online respondents was performed. No significant differences were observed regarding the perceived adequacy of telephone-based communication (*p* = 0.610) or the preferred communication method (*p* = 0.185).

By gender, women prioritized empathy and emotional contact, whereas men prioritized rapid and practical information flow.

As educational levels increased, expectations regarding information also rose; highly educated participants requested more structured, detailed, and explanatory communication.

Among younger participants, regular and clear information was more frequently perceived as helpful in alleviating concerns, and a pronounced need for daily, face-to-face updates was observed. This perceived benefit decreased with advancing age.

Regarding marital status, the need for “more information” and “seeing the patient” was most notable among married individuals, while “emotional support” was emphasized more frequently by single individuals.

Pronounced regional differences emerged: participants from the Eastern, Central Anatolian, and Southeastern regions favored face-to-face communication, whereas participants from the Mediterranean, Marmara, and Aegean regions more frequently considered telephone-based communication adequate. Relatives who were healthcare workers made more measured information requests, evaluated telephone updates as more sufficient, and emphasized “seeing the patient” as a primary need; this suggests that familiarity with clinical processes may strengthen empathetic comprehension.

The closeness of the relationship with the patient significantly affected perceived adequacy of information and communication, and responses regarding the perceived impact of physician-provided information. Participants whose child was hospitalized in the ICU were less likely to report that regular information alleviated their concerns.

As the length of stay in the intensive care unit increased, information needs shifted from quantity to quality, and demands for “emotional support,” “empathy,” and “seeing the patient” increased.

Participants who had previous experience with a relative being treated in an intensive care unit appeared more accepting of telephone-based communication. Familiarity with intensive care processes through prior exposure may reduce uncertainty regarding information delivery and influence expectations concerning the frequency and format of communication.

The demographic characteristics of the 401 participants included in the study are presented in Table 1.

Table 2 presents an analysis of the modes through which relatives receive information about patients and their perceptions regarding these methods.

A total of 304 participants responded to the optional open-ended question regarding suggestions to physicians; therefore, the results presented in this section are based on these responses.

### 3.2. Gender

The adequacy of receiving information by telephone differed significantly by gender (ꭓ^2^ = 6.00; *p* = 0.018): 32.2% of women and 43.7% of men evaluated telephone information as adequate (Table 3). The effect of physicians providing regular and clear information on concerns also differed by gender (ꭓ^2^ = 11.28; *p* = 0.004): 83.3% of women reported that this information reduced their concerns, compared with 69.5% of men. In contrast, no significant gender differences were observed in the perceived positive effect of face-to-face information, the preferred frequency of receiving updates, or the preferred method of information delivery. In both groups, daily updates and face-to-face communication were dominant preferences. The reasons for preferring a particular mode of information delivery were similar across genders, with most participants citing “clear understanding, obtaining accurate information, and being able to ask questions.” Suggestions to physicians did not differ by gender; “providing clear and understandable information,” accompanied by empathy and patience, was the most frequently expressed expectation. Information-related needs were comparable for both genders, with “more information” ranking first, followed by “seeing the patient” and “emotional support.”

### 3.3. Age

Significant associations were found between age group and information preferences (Table 4). The perceived benefit of receiving regular and clear information differed significantly according to age group and was more pronounced among younger participants (ꭓ^2^ = 13.49; *p* = 0.036). A higher percentage of younger participants preferred daily updates (ꭓ^2^ = 19.21; *p* = 0.023) and tended to favor face-to-face communication. This preference increased consistently with age and reached its highest level among participants aged 65 years and older (ꭓ^2^ = 9.86; *p* = 0.020). Across all age groups, “more information” was the primary need, followed by “seeing the patient” and “emotional support.”

### 3.4. Education

Most participants across all education levels reported a positive effect of face-to-face information (Table 5). The preferred frequency of receiving information differed significantly according to education level (*p* < 0.01): daily information requests were more common among those with lower educational attainment, whereas in the undergraduate and postgraduate groups, this preference decreased, and requests for updates “every other day” or “only when the condition changes” increased. Preferred information delivery method was also associated with education (*p* < 0.05); although face-to-face communication remained the most commonly preferred method, acceptance of telephone information increased with higher education. Suggestions to physicians were consistent across education levels, with “providing clear and understandable information” being the predominant recommendation (*p* = 0.467). Needs during the treatment process also varied significantly by education level (*p* < 0.01); “more information” was more prominent at lower education levels, while “seeing the patient” was the primary need in the postgraduate group, and the need for emotional support increased with education level.

### 3.5. Marital Status

Marital status was significantly associated with the preferred frequency of receiving information (Table 6) (ꭓ^2^ = 10.76; *p* = 0.013). While 87.7% of married participants preferred to receive information about their relative every day, 75.2% of single participants also reported preferring daily updates about their relative. A significant association was also found between marital status and preferred method of receiving information (ꭓ^2^ = 4.79; *p* = 0.029); the proportion of those preferring telephone updates was 8.1% among married individuals and 15.4% among single individuals. Needs during the treatment process differed significantly by marital status (ꭓ^2^ = 14.35; *p* = 0.002). “More information” was needed by 48.6% of married participants and 37.6% (n = 44) of single participants. The need for emotional support was expressed by 11.3% (n = 32) of married participants and 21.4% of single participants.

### 3.6. Geographic Region

The perceived adequacy of receiving information by telephone differed significantly by place of birth (ꭓ^2^ = 27.08; *p* < 0.001). Participants born in the Mediterranean, Aegean, and Marmara regions found telephone information more adequate, whereas those born in Central Anatolia and Southeastern regions rated it adequate at lower rates. Preferred frequency of receiving information also varied (*p* < 0.001): individuals from the Eastern, Southeastern, Central Anatolian, and Black Sea regions more frequently preferred daily updates, while those from Marmara, Aegean, and Mediterranean regions requested daily updates less often. Preferred information delivery method likewise differed significantly (*p* < 0.01): individuals from Eastern Anatolia, the Black Sea, and Central Anatolia preferred face-to-face communication, whereas telephone-based information was more common among participants from the Aegean, Mediterranean, Southeastern, and Marmara regions. Needs during the treatment process also varied (*p* < 0.001): “more information” was more prominent in Central Anatolia and the Black Sea; “seeing the patient” in Eastern Anatolia and the Aegean; and “emotional support” in the Mediterranean and Southeastern regions.

### 3.7. Distance

A significant relationship was found between preferred information delivery method and residing in the city where the hospital is located (ꭓ^2^ = 15.28; *p* < 0.001). Among residents of the hospital’s city, 6.4% preferred telephone communication, compared with 20.5% of those living elsewhere. Needs during the treatment process differed significantly (*p* < 0.05; Fisher’s Exact): emotional support was reported by 12.1% of those living in the hospital’s city and 19.3% of those living in other cities. The need for financial support was higher among participants residing outside the hospital’s city.

### 3.8. Employment

A statistically significant association was found between employment status and the preferred frequency of receiving information (*p* < 0.001; Fisher’s Exact). Daily updates were preferred by 79.2% of employed participants and 91.7% of non-employed participants. The proportion of participants who preferred to be informed only when there was a significant change in the patient’s condition was 6.5% among employed individuals and 3.8% among non-employed individuals. Employment status was also significantly associated with the preferred method of receiving information (χ^2^ = 4.05; *p* = 0.044). While 87.3% of employed participants preferred face-to-face information, this preference increased to 93.6% among non-employed participants.

### 3.9. Healthcare Workers

Significant differences were observed between healthcare workers and non-healthcare workers regarding perceptions of information adequacy. Half of the healthcare workers (50.6%) considered telephone information inadequate, compared with 66.2% of those outside the sector (χ^2^ = 5.76; *p* = 0.016). The adequacy of weekly information frequency also differed significantly (χ^2^ = 8.45; *p* = 0.004): 87% of healthcare workers found weekly updates sufficient, compared with 67.6% of non-healthcare workers. Preferences for information frequency varied markedly (*p* < 0.001), with healthcare workers showing a lower preference for daily updates (65.5%) and a higher preference for being informed only when the patient’s condition changed (11.6%). Regarding needs, “seeing the patient” was the primary need among healthcare workers (48.3%), whereas “receiving more information” was the dominant need among non-healthcare workers (52.2%) (*p* < 0.01). Healthcare workers also expressed a higher need for emotional support.

### 3.10. Degree of Kinship

The perceived adequacy of weekly information frequency differed significantly according to the degree of kinship (χ^2^ = 23.74; *p* < 0.001). The perceived effect of regular physician-provided information on participants’ concerns also varied according to the degree of kinship (χ^2^ = 11.28; *p* < 0.01); this effect was more pronounced among relatives whose parent, sibling, or spouse was in intensive care, and weaker among those whose child was hospitalized.

### 3.11. Duration of Treatment

As ICU stay lengthened, information preferences shifted significantly. In short-term stays (1–10 days), face-to-face communication was overwhelmingly preferred (93.8%). With longer durations, reliance on telephone information increased (χ^2^ = 8.86; *p* = 0.031). Similarly, as treatment duration increased, the need for “more information” declined, whereas the needs for “seeing the patient” and “emotional support” increased markedly (*p* < 0.001).

### 3.12. Previous ICU Experience

Participants with prior ICU experience found telephone information more adequate (χ^2^ = 8.97; *p* < 0.01) and reported a lower positive impact of face-to-face information (χ^2^ = 7.12; *p* = 0.028). However, previous ICU experience was not associated with differences in concerns reduction related to receiving regular updates from physicians (*p* = 0.743).

### 3.13. Perceived Sufficiency of Telephone-Based Information

The results of the multivariable logistic regression analysis are presented in Table 7. In the multivariable logistic regression model, male sex, residence in the city where the hospital was located, and duration of the relative’s ICU treatment were significantly associated with considering telephone-based information sufficient. Male participants had higher odds of considering telephone-based information sufficient than female participants (OR = 2.627, 95% CI: 1.399–4.932; *p* = 0.003). Participants residing in the city where the hospital was located had lower odds of considering telephone-based information sufficient compared with those living outside the hospital city (OR = 0.350, 95% CI: 0.183–0.670; *p* = 0.002). In addition, participants whose relatives had been treated in the ICU for 21–31 days had higher odds of considering telephone-based information sufficient compared with those whose relatives had been treated for 1–10 days (OR = 3.742, 95% CI: 1.420–9.864; *p* = 0.008).

## 4. Discussion

In this study, the vast majority of participants preferred face-to-face information; they reported that it affected them more positively, helped them understand the information better, and facilitated asking questions. The findings suggest that participants’ perceived trust in communication may be influenced more by eye contact and human interaction than by verbal expressions alone. These results are consistent with previous research and support trends reported in the literature. Studies conducted with families of intensive care patients have reported that both clinicians and relatives find telephone or video consultations less effective than face-to-face communication [8]. It has been reported that video or face-to-face consultations create a higher perception of care quality than telephone information. These findings indicate that the information process contains not only a cognitive but also an emotional dimension, and that face-to-face communication strengthens both the accuracy of information transfer and the perception of psychological support [9]. Importantly, preferring face-to-face communication should not be interpreted as indicating that telephone-based communication is necessarily perceived as inadequate, as these represent distinct constructs reflecting communication preference and perceived adequacy, respectively. Although most participants preferred face-to-face communication, a substantial proportion also considered telephone-based communication sufficient, indicating that these outcomes should be interpreted independently.

In this study, women prioritized emotional contact and verbal clarity in communication with patients’ relatives, whereas men gave more importance to rapid and practical information flow. For women, eye contact and empathy were fundamental components of perceived trust, while for men, fast and functional information flow was more important. This difference suggests that women may place greater importance on emotional aspects of communication, whereas men may place greater importance on rapid access to information and practical communication. However, these observations represent associations identified in the present study and should not be interpreted as inherent gender-specific characteristics.

Similarly, the literature has reported that anxiety tends to be more persistent and severe among family members of intensive care patients, particularly among female relatives [10,11]. A study conducted in Turkey also showed that depressive symptoms and hopelessness levels were higher in female first-degree relatives than in males [12]. In the multivariable logistic regression analysis, male sex, residence in the city where the hospital was located, and the duration of the relative’s ICU treatment were independently associated with considering telephone-based information sufficient. Male participants were more likely than female participants to consider telephone-based information sufficient. In contrast, participants residing in the city where the hospital was located were less likely to consider telephone-based information sufficient than those living outside the hospital city. In addition, relatives of patients who had been treated in the ICU for 21–31 days were more likely to consider telephone-based information sufficient compared with those whose relatives had been treated for 1–10 days. These findings suggest that acceptance of telephone-based communication may be influenced not only by individual characteristics but also by geographic accessibility and the stage of the intensive care process.

In the univariable analyses, participants with previous ICU experience and healthcare professionals appeared more likely to consider telephone-based communication sufficient. However, these associations were not retained after adjustment for potential confounding variables in the multivariable logistic regression model. Therefore, communication preferences observed in these groups should be interpreted with caution. Familiarity with healthcare systems and prior exposure to intensive care processes may still influence communication expectations, although this relationship was not independently supported in the adjusted analysis. Younger participants reported a greater perceived benefit from regular and clear information, suggesting age-related differences in communication expectations and information needs. While “more information” and “seeing the patient” were dominant needs among married individuals, “emotional support” and “financial assistance” were predominant among single individuals. Regional differences were observed; however, these subgroup findings should be interpreted cautiously because they originated from exploratory subgroup analyses. Individuals of Eastern–Southeastern origin preferred face-to-face and daily information, whereas those from the Marmara–Aegean–Mediterranean regions tended toward more practical telephone communication. These findings are consistent with literature reporting that satisfaction among relatives of intensive care patients is influenced by age, gender, relationship to the patient, and cultural factors [7,13]. However, the present study was not designed to determine whether the observed regional differences were attributable to cultural characteristics. These differences may also be related to socioeconomic conditions, educational background, geographic accessibility, healthcare access, or other unmeasured factors. Therefore, the observed regional differences should be interpreted as associations rather than evidence of underlying cultural determinants.

Exploratory subgroup analyses suggested that education level was associated with differences in communication preferences, perceived adequacy of information, and emotional needs; as education level increased, expectations for information and the need for emotional support also rose. These findings are broadly consistent with literature reporting higher rates of anxiety and depression among relatives with lower education [14,15], while presenting a context-specific pattern in Turkey. In contrast, studies from Iran and Lebanon did not find a significant relationship between education level and anxiety or depression symptoms [16,17]. Overall, educational level was associated with differences in information-seeking behavior and communication expectations in the present study. Highly educated individuals tend to prefer detailed and analytical information, whereas individuals with lower educational attainment may benefit from more frequent, empathetic, and supportive communication strategies.

In this study, as treatment duration lengthened, the communication preferences and expectations of relatives changed; initially, access to information and clarity were paramount, whereas over time, empathy, patience, and emotional support became more prominent. The literature also reports higher rates of self-reported concerns and depression among relatives of patients with acute illnesses compared with relatives of chronic patients [18], and an increased need for psychosocial support during prolonged intensive care processes [19]. From a practical perspective, these findings suggest that communication strategies in ICUs should not follow a uniform approach. Communication preferences were associated with age, gender, educational level, previous ICU experience, and regional characteristics. Tailoring the frequency, format, and content of information to family characteristics may better address relatives’ communication preferences, improve the perceived adequacy of communication, and strengthen perceived trust between healthcare professionals and relatives.

## 5. Limitations

This study has several limitations. Participants were recruited using a non-probability sampling strategy, and data were collected through both face-to-face interviews and an online questionnaire. Although identical questionnaires and standardized administration procedures were used for both methods, differences in accessibility, willingness to participate, and respondent characteristics may have introduced selection bias. Therefore, caution should be exercised when generalizing the findings to all relatives of intensive care patients.

Although participants were recruited from different regions of Turkey, the use of a non-probability sampling strategy may have limited the representativeness of the study population and the regional and sociodemographic diversity of participants. Therefore, the observed regional and sociodemographic differences should be interpreted as associations within the study sample rather than findings that can be generalized to all relatives of intensive care patients in Turkey. Individuals who agreed to participate may have differed systematically from those who declined participation, introducing additional selection bias. The data were based on self-reported perceptions and experiences, which may have been influenced by recall bias, emotional state, or social desirability bias. Therefore, the findings reflect participants’ subjective evaluations rather than objectively measured psychological outcomes.

The questionnaire was specifically developed for this study. Although its face validity was reviewed by experts and pilot-tested, constructs such as satisfaction, trust, anxiety, and communication expectations were not assessed using previously validated psychometric instruments. Accordingly, the findings should be interpreted as participants’ self-reported perceptions and preferences rather than validated measures of psychological constructs or formal psychological assessments.

In addition, the cross-sectional design precludes causal inference. Associations observed between sociodemographic characteristics and communication preferences should therefore not be interpreted as causal relationships. Future multicenter studies using representative sampling methods and validated measurement instruments are needed to confirm and extend these findings.

Finally, multiple exploratory subgroup analyses were performed without formal adjustment for multiple comparisons. Therefore, these subgroup findings should be interpreted cautiously and considered hypothesis-generating rather than definitive. Future studies are warranted to confirm these observations.

## 6. Conclusions

In conclusion, face-to-face communication was the preferred method of information delivery for the vast majority of participants and was more frequently perceived as promoting trust and understanding. Communication preferences varied according to demographic, educational, experiential, and regional characteristics, suggesting that a uniform communication strategy may not adequately address the needs of all families. In multivariable analysis, male sex, residence outside the city where the hospital was located, and having a relative hospitalized in the intensive care unit for 21–31 days were independently associated with considering telephone-based communication sufficient. These findings suggest that communication strategies in intensive care units should be tailored according to relatives’ characteristics, accessibility to the hospital, and the patient’s treatment course rather than relying on a uniform approach. Future prospective multicenter studies are needed to determine whether individualized communication strategies improve perceived adequacy of information and communication outcomes.

## Figures and Tables

**Table 1:** Sociodemographic characteristics of the participants.

Variable	Level	n	%
Gender	Female	227	56.6
	Male	174	43.4
	Total	401	100.0
Age group	18–34	131	32.7
	35–49	126	31.4
	50–64	109	27.2
	≥65	35	8.7
	Total	401	100.0
	Mean ± SD/Median	43.4 ± 14.8/44	
	Minimum–Maximum	18–75	
Education level	Illiterate	3	0.7
	Primary education	104	25.9
	High school	114	28.5
	Undergraduate	144	35.9
	Postgraduate	36	9.0
	Total	401	100.0
Marital status	Married	284	70.8
	Single	117	29.2
	Total	401	100.0

**Table 2:** Preferences for information acquisition, perceived adequacy, and effects on concerns levels among all participants.

Variable	Level	n	%
Effect of receiving face-to-face information about the patient’s condition	Positive	355	88.5
	No effect	37	9.3
	Negative	9	2.2
	Total	401	100.0
Adequacy of receiving information by telephone	Adequate	149	37.2
	Not adequate	252	62.8
	Total	401	100.0
Effect of the physician providing regular and clear information on concerns	Decreases	310	77.3
	No effect	71	17.7
	Increases	20	5.0
	Total	401	100.0
Preferred frequency of receiving information	Every day	337	84.1
	Every other day	37	9.2
	1–2 times per week	5	1.2
	Only when there is a significant change in the patient’s condition	22	5.5
	Total	401	100.0
Preferred method of receiving information	Face-to-face	360	89.8
	Telephone	41	10.2
	Total	401	100.0
Main reason for preferred method of information acquisition	I understand better; I can obtain clear information and ask questions	209	52.1
	Seeing the doctor face to face makes me feel better; it increases perceived trust	120	30.3
	It is easy, quick, and effortless	53	13.1
	Other	19	4.5
	Total	401	100.0
Suggestions to physicians regarding informing relatives	Provide information clearly and understandably	164	53.9
	Allow relatives to see the patient more frequently	33	10.9
	Show empathy and provide information patiently and sincerely	94	30.9
	Pay more attention to the patient	6	2.0
	Other	7	2.3
	Total	304	100.0

**Table 3:** Perceived adequacy of information methods according to the gender of patients’ relatives.

Variable	Level	Gender of Patient’s Relative			*p* * (χ^2^)
		**Female**		**Male**	
		**n**	**%**	**n**	**%**
How do you receive information about your relative’s condition?	Face-to-face	136	59.9	90	51.7
	By phone	12	5.3	9	5.2
	Both	79	34.8	75	43.1
	Total	227	100.0	174	100.0
How do you evaluate the effect of receiving face-to-face information about your relative’s condition?	Positive	204	89.9	151	86.8
	No effect	20	8.8	17	9.8
	Negative	3	1.3	6	3.4
	Total	227	100.0	174	100.0
How do you evaluate the adequacy of receiving information by phone about your relative’s condition?	Adequate	73	32.2	76	43.7
	Inadequate	154	67.8	98	56.3
	Total	227	100.0	174	100.0
How many days per week are you informed about your relative’s condition?	1–2 days	12	5.4	11	6.5
	3–4 days	19	8.6	20	11.8
	5 days or more	190	86.0	138	81.7
	Total	221	100.0	169	100.0
How do you evaluate the adequacy of this frequency?	Adequate	115	71.0	92	74.8
	Inadequate	47	29.0	31	25.2
	Total	162	100.0	123	100.0
How does receiving regular and clear information from the physician affect your concerns level?	Decreases	189	83.3	121	69.5
	No effect	28	12.3	43	24.8
	Increases	10	4.4	10	5.7
	Total	227	100.0	174	100.0

* Chi-square.

**Table 4:** Evaluation of the methods and adequacy of obtaining information about the health status of relatives according to age.

Variable	Level	18–34 n (%)	35–49 n (%)	50–64 n (%)	≥65 n (%)	*p* * (χ^2^)
How do you evaluate the effect of receiving face-to-face information about your relative’s condition?	Positive	112 (85.5)	110 (87.3)	100 (91.7)	33 (94.2)	0.207 (8.45)
	No effect	18 (13.7)	12 (9.5)	6 (5.5)	1 (2.9)	
	Negative	1 (0.8)	4 (3.2)	3 (2.8)	1 (2.9)	
	Total	131 (100)	126 (100)	109 (100)	35 (100)	
How do you evaluate the adequacy of receiving information by phone about your relative’s condition?	Adequate	45 (34.4)	51 (40.5)	39 (35.8)	14 (40.0)	0.742 (1.25)
	Inadequate	86 (65.6)	75 (59.5)	70 (64.2)	21 (60.0)	
	Total	131 (100)	126 (100)	109 (100)	35 (100)	
How many days per week are you informed about your relative’s condition?	1–2 days	14 (11.5)	6 (4.8)	2 (1.9)	1 (2.9)	<0.001 (28.23)
	3–4 days	22 (18.0)	7 (5.6)	10 (9.3)	0 (0.0)	
	≥5 days	86 (70.5)	112 (89.6)	96 (88.8)	34 (97.1)	
	Total	122 (100)	125 (100)	108 (100)	35 (100)	
How do you evaluate the adequacy of this frequency?	Adequate	62 (72.1)	81 (80.2)	52 (66.7)	21 (72.4)	0.115 (5.92)
	Inadequate	24 (27.9)	20 (19.8)	26 (33.3)	8 (27.6)	
	Total	86 (100)	101 (100)	78 (100)	29 (100)	
How does receiving regular and clear information from the physician affect your concerns level?	Decreases	111 (84.7)	99 (78.5)	73 (67.0)	27 (77.1)	0.036 (13.49)
	No effect	15 (11.5)	21 (16.7)	27 (24.8)	8 (22.9)	
	Increases	5 (3.8)	6 (4.8)	9 (8.2)	0 (0.0)	
	Total	131 (100)	126 (100)	109 (100)	35 (100)	

*: Chi-square.

**Table 5:** Information preferences and recommendations by educational level.

Variable	Level	Illiterate n (%)	Primary School n (%)	High School n (%)	Bachelor’s n (%)	Postgraduate n (%)	*p* * (χ^2^)
How do you evaluate the effect of receiving face-to-face information about your relative’s condition?	Positive	3 (100)	98 (94.2)	98 (86.0)	126 (87.5)	30 (83.3)	0.101 **
	No effect	0 (0)	3 (2.9)	12 (10.5)	17 (11.8)	5 (13.9)	
	Negative	0 (0)	3 (2.9)	4 (3.5)	1 (0.7)	1 (2.8)	
	Total	3 (100)	104 (100)	114 (100)	144 (100)	36 (100)	
How do you evaluate the adequacy of receiving information by phone about your relative’s condition?	Adequate	2 (66.7)	39 (37.5)	38 (33.3)	54 (37.5)	16 (44.4)	0.616 (2.66)
	Inadequate	1 (33.3)	65 (62.5)	76 (66.7)	90 (62.5)	20 (55.6)	
	Total	3 (100)	104 (100)	114 (100)	144 (100)	36 (100)	
How many days per week are you informed about your relative’s condition?	1–2 days	0 (0)	1 (1.0)	9 (8.3)	11 (7.9)	2 (5.6)	0.041 (16.12)
	3–4 days	0 (0)	5 (4.9)	11 (10.1)	21 (15.1)	2 (5.6)	
	≥5 days	3 (100)	97 (94.1)	89 (81.6)	107 (77.0)	32 (88.8)	
	Total	3 (100)	103 (100)	109 (100)	139 (100)	36 (100)	
How do you evaluate the adequacy of this frequency?	Adequate	1 (100)	55 (69.6)	55 (63.2)	73 (78.5)	23 (92.0)	0.027 (10.94)
	Inadequate	0 (0)	24 (30.4)	32 (36.8)	20 (21.5)	2 (8.0)	
	Total	1 (100)	79 (100)	87 (100)	93 (100)	25 (100)	
How does receiving regular and clear information from the physician affect your concerns level?	Decreases	2 (66.7)	79 (76.0)	89 (78.1)	117 (81.3)	23 (63.9)	0.073 (14.36)
	No effect	1 (33.3)	17 (16.3)	21 (18.4)	19 (13.2)	13 (36.1)	
	Increases	0 (0)	8 (7.7)	4 (3.5)	8 (5.6)	0 (0)	
	Total	3 (100)	104 (100)	114 (100)	144 (100)	36 (100)	
How frequently would you prefer to receive information about your relative?	Every day	3 (100)	94 (90.4)	105 (92.1)	112 (77.8)	23 (63.9)	0.004
	Every other day	0 (0)	5 (4.8)	5 (4.4)	19 (13.2)	8 (22.2)	
	1–2 times per week	0 (0)	1 (1.0)	1 (0.9)	3 (2.1)	0 (0)	
	When there is a significant change in condition	0 (0)	4 (3.8)	3 (2.6)	10 (6.9)	5 (13.9)	
	Total	3 (100)	104 (100)	114 (100)	144 (100)	36 (100)	
Which method would you prefer to receive information about your relative?	Face-to-face	3 (100)	101 (97.1)	100 (87.7)	122 (84.7)	34 (94.4)	0.012
	By phone	0 (0)	3 (2.9)	14 (12.3)	22 (15.3)	2 (5.6)	
	Total	3 (100)	104 (100)	114 (100)	144 (100)	36 (100)	
Do you have any recommendations for physicians regarding informing patients’ relatives?	Provide information clearly and understandably	2 (100)	39 (50.0)	53 (57.0)	61 (54.5)	9 (47.4)	0.467
	Allow patients to be seen more frequently	0 (0)	12 (15.4)	11 (11.8)	8 (7.1)	2 (10.5)	
	Show empathy and provide information patiently and sincerely	0 (0)	24 (30.8)	26 (28.0)	39 (34.8)	5 (26.3)	
	Show more interest in the patient	0 (0)	2 (2.6)	1 (1.1)	1 (0.9)	2 (10.5)	
	Other	0 (0)	1 (1.3)	2 (2.2)	3 (2.7)	1 (5.3)	
	Total	2 (100)	78 (100)	93 (100)	112 (100)	19 (100)	

*: Chi-square, **: Fisher-exact.

**Table 6:** Evaluation of participants’ preferences for receiving information according to marital status.

Variable	Level	Marital Status		χ^2^	*p* *
		**Married**		**Single**	
		**n**	**%**	**n**	**%**
How frequently would you prefer to receive information about your relative?	Every day	249	87.7	88	75.2
	Every other day	22	7.7	15	12.8
	1–2 times per week	2	0.7	3	2.6
	When there is a significant change in condition	11	3.9	11	9.4
	Total	284	100.0	117	100.0
Which method would you prefer to receive information about your relative?	Face-to-face	261	91.9	99	84.6
	By phone	23	8.1	18	15.4
	Total	284	100.0	117	100.0

*: Chi-square.

**Table 7:** Multivariable Logistic Regression Analysis of Factors Associated with Considering Telephone-Based Communication Sufficient.

Predictor	Estimate	SE	Z	*p*	OR	95% CI	
						Lower	Upper
Gender							
Male—Female	0.966	0.321	3.005	0.003	2.627	1.399	4.932
Region							
Eastern Anatolia—Mediterranean	−0.866	0.847	−1.022	0.307	0.420	0.080	2.213
Aegean—Mediterranean	−0.214	0.859	−0.250	0.803	0.807	0.150	4.349
Southeastern Anatolia—Mediterranean	−1.189	1.124	−1.057	0.290	0.305	0.034	2.759
Central Anatolia—Mediterranean	−1.028	0.743	−1.383	0.167	0.358	0.083	1.535
Black Sea—Mediterranean	0.075	0.725	0.103	0.918	1.078	0.260	4.466
Marmara—Mediterranean	0.031	0.833	0.037	0.971	1.031	0.202	5.273
Residence in the city where the hospital was located							
Yes—No	−1.049	0.331	−3.170	0.002	0.350	0.183	0.670
Healthcare-sector employment							
Yes—No	0.651	0.334	1.947	0.052	1.917	0.996	3.690
Duration of relative’s ICU treatment							
11–20 days—1–10 days	0.456	0.364	1.255	0.210	1.578	0.774	3.220
21–31 days—1–10 days	1.320	0.495	2.669	0.008	3.742	1.420	9.864
31+—1–10 days	0.770	0.541	1.425	0.154	2.161	0.749	6.234
Previous ICU treatment experience involving another relative							
Yes—No	0.561	0.407	1.378	0.168	1.752	0.789	3.893

Note. The outcome was coded as sufficient versus insufficient telephone-based information. VIF values ranged from 1.043 to 1.097 and tolerance values from 0.911 to 0.958, indicating no multicollinearity. The overall model was statistically significant, χ^2^(13) = 58.247, *p* < 0.001, McFadden’s R^2^ = 0.178. SE, standard error; OR, odds ratio; CI, confidence interval.

## Data Availability

The raw data supporting the conclusions of this article will be made available by the authors on request.

## Supplementary Materials

The following supporting information can be downloaded at https://www.mdpi.com/article/10.3390/healthcare14132026/s1, File S1: Survey Questions, File S2: Survey screenshot, File S3: Patient Relative Answers, File S4: Sample Size and Power Analysis.

## Abbreviations

ICU	Intensive care unit
