# Supplementary material for: Informing Patient Relatives in Intensive Care Units… Face to Face or by Phone?
Source: Healthcare (Basel). 2026 Jul 7;14(13):2026. doi: 10.3390/healthcare14132026 (PMC13361816; doi:10.3390/healthcare14132026)
Supplement: Supplementary file 1 [file healthcare-14-02026-s001.zip › Supplementary Material File S1 (SURVEY QUESTIONS).pdf]

**1.Do you voluntarily agree to participate in this study and answer the questions of your own free will?**

I agree

I do not agree

**2.What is your age?**

**3.What is your gender?**

Male

Female

**4.What is your level of education?**

No formal education

Primary school education

High school graduate

Bachelor’s degree

Postgraduate

**5.What is your marital status?**

Married

Single

**6.What is your relationship to the patient admitted to the intensive care unit (ICU)?**

Parent (mother/father)

Sibling

Spouse

Child

Other

**7.Have you previously had a relative admitted to an ICU?**

Yes

No

**8.If yes, does prior experience with the ICU process reduce your concerns?**

Yes

No

**9.How many days has your relative been admitted to the ICU?**

1–10 days

11–20 days

21–30 days

More than 30 days

**10.How many days per week are you informed about your relative's condition? Do you consider this frequency sufficient?**

**1–2 days**

**3–4 days**

**≥5 days**

**Adequate / Inadequate**

**11.Does receiving regular and clear information from the physician affect your level of concerns?**

Reduces it

Increases it

Has no effect

**12.How do you receive information about your patient from the physician?**

Face-to-face

By telephone

Both face-to-face and by telephone

**13.Which method do you prefer for receiving information about your patient?**

Face-to-face

By telephone

**14.Please explain the reason for your preferred method of receiving information. ( open-ended questions)**

**15.How many days per week would you like to receive information about your patient in the ICU?**

Every day

Every other day

1–2 times per week is sufficient

Only when there is a significant change in the patient's condition

**16.As a relative of an ICU patient, what is your greatest need during the treatment process?**

Receiving more information

Receiving more emotional support

Seeing my patient in person

Financial support

**17.Is receiving information about your patient via telephone sufficient for you?**

Yes

No

**18.Does receiving information face-to-face make you feel better?**

Yes

No

The method of receiving information does not affect my feelings

**19.Please provide any suggestions you have for physicians regarding informing patient relatives. ( open-ended questions)**

**20.Do you consider yourself a social person?**

Yes

No

**21.Please select the option that best describes your employment status.**

I work in the public sector

I work in the private sector

I am not currently employed

**22.Do you work in the healthcare sector?**

Yes

No

**23.In which geographical region were you born?**

Eastern Anatolia

Southeastern Anatolia

Central Anatolia

Marmara Region

Aegean Region

Mediterranean Region

Black Sea Region

**24.Do you live in the same city where your relative is admitted to the ICU?**

Yes

No

**25.Do you reside close enough to visit the hospital where your patient is being treated whenever and as often as you wish?**

Yes

No
