# Supplementary figures and images for: Informing Patient Relatives in Intensive Care Units… Face to Face or by Phone?
Source: Healthcare (Basel). 2026 Jul 7;14(13):2026. doi: 10.3390/healthcare14132026 (PMC13361816; doi:10.3390/healthcare14132026)

# Supplementary Materials S4. Sample size and power analysis

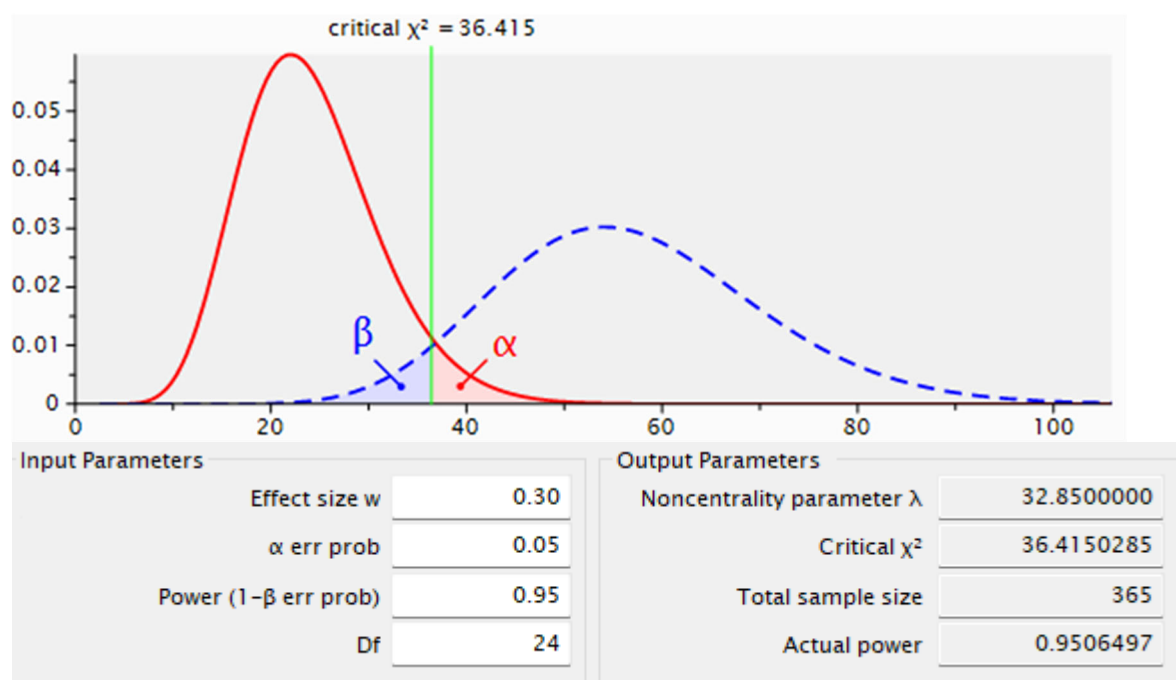

Supplement: Supplementary file 1 [file healthcare-14-02026-s001.zip › Supplementary Material File S4 (Sample Size and Power Analysis).pdf]
